# Early Detection of Atrial Fibrillation Based on ECG Signals

**DOI:** 10.3390/bioengineering7010016

**Published:** 2020-02-13

**Authors:** Nuzhat Ahmed, Yong Zhu

**Affiliations:** 1 Bioengineering 4+1 Program, Wilkes University, Wilkes-Barre, PA 18701, USA; nuzhat.ahmed@wilkes.edu; 2 Mechanical Engineering, Wilkes University, Wilkes-Barre, PA 18701, USA

**Keywords:** atrial fibrillation (AF), ECG signal, biosensor, heart health monitoring, low-cost device

## Abstract

Atrial fibrillation, often called AF is considered to be the most common type of cardiac arrhythmia, which is a major healthcare challenge. Early detection of AF and the appropriate treatment is crucial if the symptoms seem to be consistent and persistent. This research work focused on the development of a heart monitoring system which could be considered as a feasible solution in early detection of potential AF in real time. The objective was to bridge the gap in the market for a low-cost, at home use, noninvasive heart health monitoring system specifically designed to periodically monitor heart health in subjects with AF disorder concerns. The main characteristic of AF disorder is the considerably higher heartbeat and the varying period between observed R waves in electrocardiogram (ECG) signals. This proposed research was conducted to develop a low cost and easy to use device that measures and analyzes the heartbeat variations, varying time period between successive R peaks of the ECG signal and compares the result with the normal heart rate and RR intervals. Upon exceeding the threshold values, this device creates an alert to notify about the possible AF detection. The prototype for this research consisted of a Bitalino ECG sensor and electrodes, an Arduino microcontroller, and a simple circuit. The data was acquired and analyzed using the Arduino software in real time. The prototype was used to analyze healthy ECG data and using the MIT-BIH database the real AF patient data was analyzed, and reasonable threshold values were found, which yielded a reasonable success rate of AF detection.

## 1. Introduction

Cardiovascular diseases are considered to be the leading cause of death worldwide. In fact, CVDs are the number one cause of death globally, accounting for about 17.3 million deaths per year, and this number is expected to grow to more than 23.6 million by 2030 [1]. This is a group of diseases which includes coronary artery diseases (CAD) such as stable angina, unstable angina, and myocardial infarction (MI) which is commonly known as a heart attack, and high blood pressure, diabetes, smoking, lack of exercise or physical activities, obesity, high blood cholesterol, poor diet, and excessive amount of alcohol consumption etc. increase the risk of coronary artery diseases to a great extent [2]. People who have been diagnosed with cardiovascular disease as well as those who are at high risk for it because of the presence of one or multiple risk factors—hypertension, diabetes, hyperlipidemia, or other various established diseases—need early detection in order to receive proper treatment [3].

Atrial fibrillation (AF) is the most common type of cardiac arrhythmia, which is a major healthcare challenge. AF is associated with a high risk of cardiovascular events, including cardiovascular mortality, major cardiovascular events, heart failure, ischemic heart disease, sudden cardiac death, as well as stroke [4]. This disorder can be diagnosed by feeling the pulse which can be inaccurate sometimes due to increased heart rate. Hence, the diagnosis can be best confirmed by using an electrocardiogram (ECG). It is a more sophisticated and one of the frequently used methods to measure the heart rate utilizing electronic techniques. The correct blood flow in the heart depends on an appropriate contraction of the cardiac muscle and that is due to the generation and transmission of electrical impulses. It is this biological electrical activity that can be recorded and analyzed using the ECG signal which is very useful in the diagnosis of a significant amount of cardiac abnormalities [5].

ECG is the best way to detect AF disorder, however, ECG testing and analyzing the reading is not a conventional or a frequent process that can be performed at home using a typical ECG apparatus as first, it is very expensive—the national ECG procedure pricing is in the range of $550–$3300 and the average cost is about $1750 [6]. Second, it is not possible to conduct the testing process and interpret the reading by an individual with little or no knowledge of ECG. Therefore, this research project proposes the development of a very low cost, user friendly, and portable heart health monitoring device—a system that will be able to capture the electrical signal originating from the heart using a bio potential ECG sensor and the microcontroller will analyze the heart rate using the ECG signal in real time. The device will use a straightforward yet effective enough algorithm that will detect certain abnormalities, followed by creating an early warning for potential heart issues. This device will comprise of electronics, computer, and signal processing tools to provide better diagnosis of cardiac pathologies.

AF disorder is an abnormal heart rate depicted by instantaneous and irregular beating of the atria. It often starts as momentary periods of abnormal beating and eventually becomes longer and possibly constant over time. This disorder can increase the risk of stroke, heart failure, and various other heart-related complications. During AF, the upper two chambers of the heart (the atria) beat chaotically and irregularly. The rhythm is more likely out of coordination with the lower two chambers of the heart which are called ventricles [7]. AF is a result of poor blood supply and flow throughout the body and this disruption causes blood clot formation in the heart that eventually circulate to other organs and lead to blocked blood flow which is known as Ischemia [7]. When these clots travel to the brain and create a blockage in a blood vessel, the most common and damaging type of stroke occurs and about 20% of this type of stroke results directly from AF [4]. It is also found that apart from many other different symptoms and repercussions, people with AF disorder are five times more likely to suffer a stroke [8]. Furthermore, strokes that results from AF are more severe and cause greater disability than strokes in patients without AF.

AF symptoms commonly include heart palpitations, shortness of breath, weakness, dizziness, fainting, fatigue, and chest pain. It is estimated that about half of AF patients remain undetected and the reason is patients often ignore the symptoms and are not aware of the fact that the symptoms they experience could be a sign of potential cardiac abnormalities [9]. This ignorance or lack of proper awareness can have lasting damage on the heart as the longer the heart is out of rhythm, the greater potential the shape will change and cause it to work harder, and eventually will lead to congestive heart failure. The heart rate in AF may range from 100 to 175 beats per minute whereas the normal range for a heart rate is 60 to 100 beats per minute [7]. AF disorder itself is not life threatening but as mentioned earlier, most of the times the symptoms are ignored, unnoticed, and therefore remain undetected. This could lead to fatal and debilitating consequences which include heart failure, stroke, poor mental health, reduced quality of life, and death. AF is generally intermittent and innumerable, therefore people with AF disorder have no or nonspecific symptoms. Consequently, this combination makes the detection and diagnosis very difficult. Most of the time AF is not apparent until a person encounters a serious health complication and goes for the treatment. Additionally, those patients who do experience some symptoms of AF are not always diagnosed immediately due to the lack of awareness. Moreover, it is found that approximately three-quarters of the world’s deaths from CVDs occur in low-income and middle-income countries. People in those countries often do not retain the consolidated primary health care benefit for early detection and treatment of cardiac abnormalities in comparison to people in high-income countries. Often people in low-income and middle-income countries with the potential risk factors of CVDs have less access to effectual and unprejudiced health care services and investigations [3]. As a result, there is a remarkable possibility that many people in low-income and middle-income countries will be detected late or even remain undetected for AF disorder. Therefore, early detection of potential AF using a low-cost device is crucial so that proper investigation and treatment can be performed. Since symptoms related to AF disorder can come and go, therefore, the proposed device will be suggested to be used if the symptoms seem to be consistent and persistent.

Some background research was carried out to study the existing devices and previous research work regarding heart health monitoring systems. A real-time health monitoring system was developed to create a remote monitoring diagnostic system for remote cardiac patients [10]. The system used a smartphone and wearable sensors that would analyze heart rate, blood pressure, body and skin temperature at the same time and will help early detection of various cardiac diseases through an alarm system based on upper and lower threshold values.

A health monitoring system study proposes a portable monitoring system that monitors and analyzes the heart and notifies about the status [11]. This system consists of various types of sensors and the collected information is then transferred wirelessly to a smartphone. This particular system is distinct from other systems because it can personalize the monitoring and also has mechanisms to locate the user. A beat detection and classifier algorithm and a VT/VF detection algorithm for the smartphone were used to detect life threatening arrhythmias.

Another heart rate monitoring system focuses on developing a system that determines the heartbeat rate per minute and then sends a short message alert to the mobile phone [12]. This system was based on principle of photo plethysmography (PPG)—a noninvasive method of measuring the changes in blood volume in tissue using a light source and detector.

HeartSaver, a mobile cardiac monitoring system for auto-detection of atrial fibrillation, myocardial infarction, and atrio-ventricular block was developed [13]. It incorporates four different modern technologies—electronics, wireless communication, computer, and information technologies—to develop a low-cost portable device that will monitor a patient’s ECG. A microcontroller converted the analog heart signals extracted from skin and filtered using ECG filter unit to digital signal and sent to a phone via a wired link. Software called CellTrack and MIDlet were used to send a text when microcontroller sent a signal to the phone indicating a cardiac emergency.

The intelligent telecardiology study attempted to develop an intelligent wireless system with a built-in abnormal ECG detection mechanism and an alert expert system for the early detection of cardiac disorders [14]. This system consists of a three-lead wireless ECG device, a Java-based expert system application, and a web-based monitoring platform.

There are a number of heart health monitoring devices on the market such as the AliveCor that shows positive benefit, especially for part time AF patients. This device is costly and will not detect those with who experience no symptoms for AF disorder. There are also different tools available now that can be used outside of the hospital area for AF detection, for example Single Lead, Holter Monitoring, Mobile Telemetry Monitoring, and Implantable Loop Recorders. With spot single-lead ECG devices like the AliveCor, the patient has to recognize symptoms, record, and share with their clinician. Holter monitoring device is used for a period of 24 h. This device costs about $2000–3000 and is only an ECG recording device with no detection or telemetry capability. Mobile telemetry monitors can be costly and bulky. Finally, the implantable loop recorder is costly and does not produce any immediate feedback for the patient and has connectivity requirements [15,16]. Therefore, in a nutshell, it can be summarized that existing heart health monitoring systems are either too complicated or require special expensive devices that are to be paired with extensive signal processing to obtain better quality results.

A low-cost, self-operated heart health monitoring system is crucial to reduce the cost of medical checkup and the anxiety of people with known or at risk of cardiovascular problems and also to help them to obtain proper treatment on time. Hence, this proposed research work will be feasible and nearly as accurate in early detection of potential AF disorder as well as a host of other diseases in real time, with an objective of diminishing the gap in the market for a low-cost, at home use, noninvasive heart health monitoring system specifically designed to periodically monitor heart health in subjects with AF disorder concerns.

## 2. Materials and Methods

**Figure 1:**
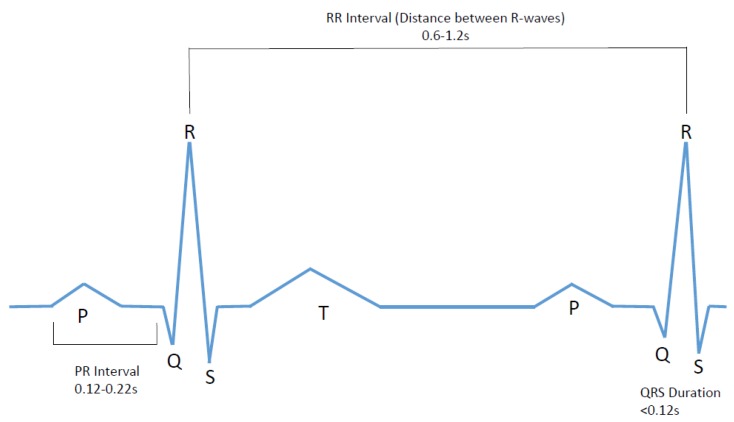
Normal electrocardiogram (ECG) signal with wave durations and intervals.

An ECG signal represents the electrical activity of the four chambers of the heart. This signal is a series of a P wave, QRS complex, and a T wave as shown in Figure 1.

The P wave indicates atrial depolarization. The PR interval begins at the beginning of the P wave and ends at the beginning of the QRS complex. The PR interval represents the time during which a depolarization wave travels from the atria to the ventricles. The QRS interval stipulates ventricular depolarization and atrial repolarization. It contains three deflections: Q wave, R wave, and the S wave [15,16,17].

The main characteristic of AF disorder is the irregular rhythm of the heartbeat or more specifically when a varying period is observed in ECG signal between R–R peaks. Therefore, this research project focuses on developing an affordable and user-friendly diagnostic ECG system that will study the ECG signal for both normal and AF, followed by analyzing heart rate variations and comparing the result with the predetermined values to create an alert in the case of cardiac abnormalities. The system is required to collect data for about 60 s and during this time subject will be required to abstain from any kind of movement or speaking and maintain regular breathing. After that the data is to be analyzed in terms of counting the heart beats per minute, determining the R peak, and then finding the RR interval in real time. The device will contribute towards possible AF detection by confirming two decision making factors—considerably higher beats per minute (BPM) (10–175), heartbeat interval variations (inconsistent and not within 0.6–1.2 s range).

**Figure 2:**
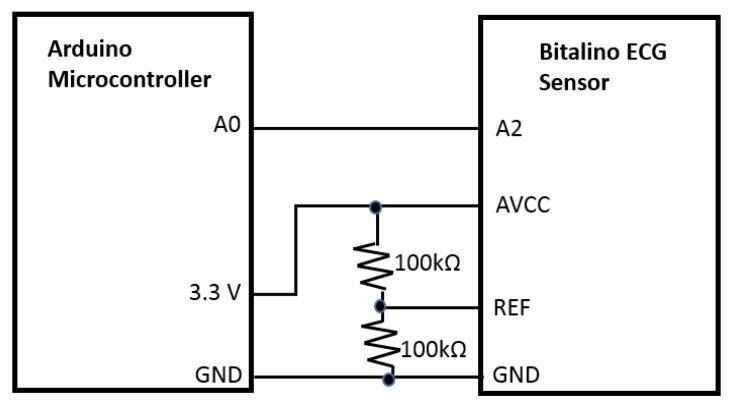
Circuit diagram of the prototype.

The system will function using the Bitalino ECG sensor that apprehends the electrical signal originated from the heart. We utilized Bitalino bio-signal sensor kit to collect ECG signals because it is a low cost, noninvasive commercial device. The bandwidth is 0.5–40 Hz to guarantee the accuracy and resolution of ECG signals. The acquired voltage can be visualized by the software OpenSignals first, which enables real-time data acquisition and offline browsing. For ECG acquisition using Bitalino, two sampling frequencies, 100 and 1000 Hz, are available. A recent study [18] shows “a close similarity between data acquired by the BITalino and by the reference device.” The output of the sensor, the extracted heart signal, was sent to the central processing unit, Arduino microcontroller port, for monitoring and counting purposes. The data acquisition unit was constructed by building a circuit where the Bitalino sensor (AVCC) was wired to the 3.3 V power supply and the ground pin of the Arduino microcontroller. The output pin of the Bitalino sensor (A2) was connected to one of the analog ports of the Arduino board for the signal processing. Additionally, 2 × 100 KΩ resistors were used as voltage divider for the sensor reference (REF) (Figure 2). The Arduino microcontroller was then connected to the computer with the USB cable to connect the hardware with the Arduino software.

**Figure 3:**
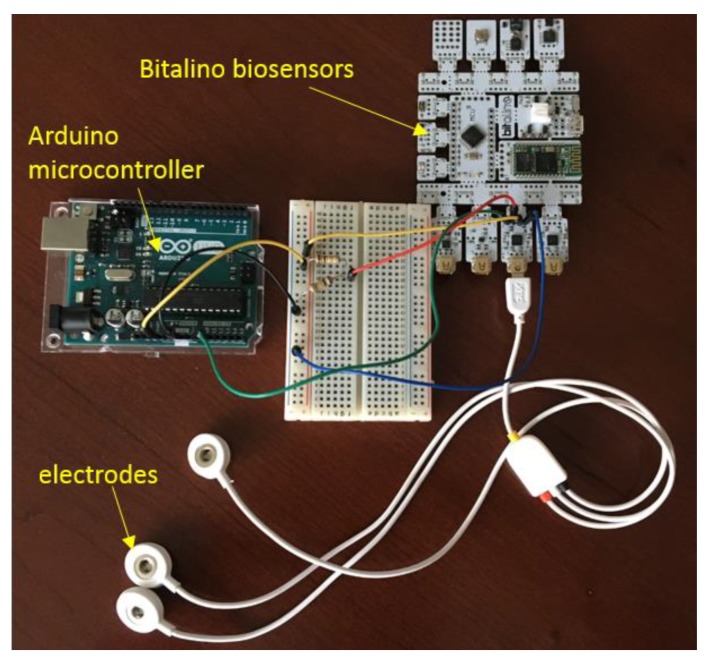
Prototype experimental setup.

The first part of the Arduino algorithm was developed to extract and plot the raw ECG signal and to convert the signal using transfer function and process it with digital filtering method. The second part of the algorithm determined the heart beats per minute of the healthy ECG signal, followed by the RR interval of the ECG signal. The third part of the algorithm was developed to compare the result acquired from second part with the normal values which was included in the algorithm as the threshold value to show the alert for any potential cardiac events. The hardware construction and the electrodes are shown in Figure 3 based on a preliminary work carried out previously [19].

The data for healthy ECG signal was first collected by self-testing, extracting ECG data by using Bitalino ECG sensor, Arduino microcontroller, and Arduino software. The AF ECG signal data was obtained from the MIT-BIH Atrial Fibrillation Database [20,21]. Matlab and Arduino software were used for the further signal processing and analysis in order to achieve the smooth and better quality ECG signal by removing the noise. Afterwards, using the data obtained from the serial monitor of the Arduino and the online database, Matlab and Arduino algorithms were developed to determine the heart beats per minute. The BPM counter function was used where a specific timer was set during which each peak of the ECG signal was counted. The next step was to determine prominent R peak of the ECG signal as well as samples, study the interval between R waves, and detect the consistency. For this, about 60 time intervals were collected to perform a statistical analysis in real time. The process is discussed in detail in Section 3. After that, AF detection success rate was also determined using multiple human subjects ECG data.

## 3. Results

**Figure 4:**
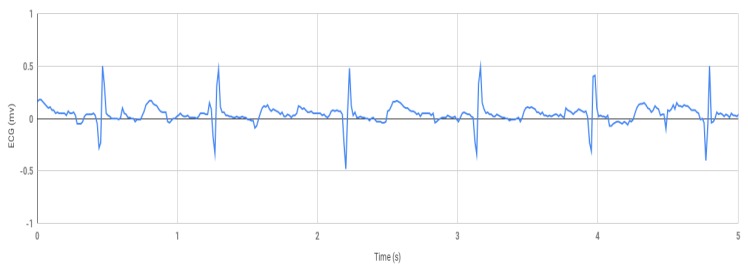
Consistent ECG signal obtained from the Arduino serial monitor.

The first part of this research work involves the healthy ECG data shown in Figure 4, which was obtained by using the prototype experimental setup shown in Figure 3. Using the Arduino software, the data was collected from Arduino serial monitor and then was plotted in Excel (Figure 4). During testing, the negative electrode on the left palm and the positive electrode on the right palm produced inconsistent signal but decent results were obtained within 1.5 to −1.5 mv shown in Figure 4 when the positive electrode was placed on the right wrist, and the negative on the left wrist.

**Figure 5:**
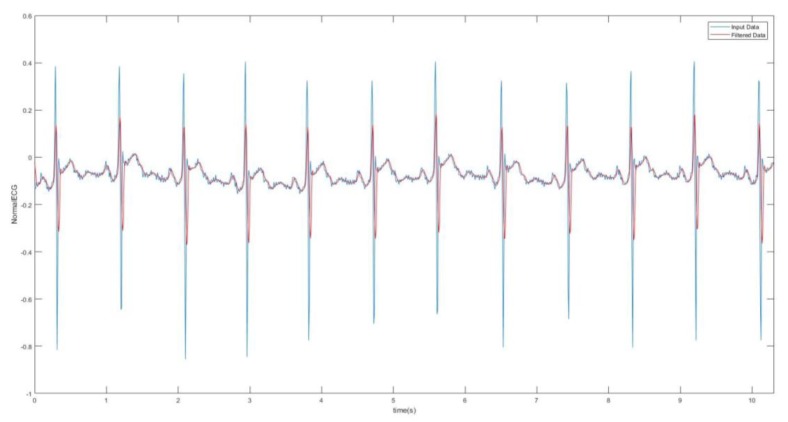
Healthy filtered ECG.

Digital filtering methods were incorporated which involved taking the RMS value of the signal. The RMS value function takes the square root of the squared mean of a set of values, which was completed by using a loop reiterated 10 times. This means the Arduino code with the RMS value function plotted one point for every 10 that it integrated. The RMS value seemed to slightly smoothen the red curve. Another method that was used to smoothen the signal significantly was the moving-average filter where the filter function slides a window of size 5 along the data, computing averages of the data contained in each window. The moving average of the data and the original data were plotted against time in Figure 5. This filtering was done as an attempt of smoothing the ECG signal in order to determine the presence of the P wave in the ECG signal. This is because in AF ECG usually there are no P waves. However, for the AF detection, studying the P wave can be tricky as the baseline of the ECG in AF is a wavy chaotic baseline that can sometimes appear like the P wave which are just random changes in the chaotic baseline.

**Figure 6:**
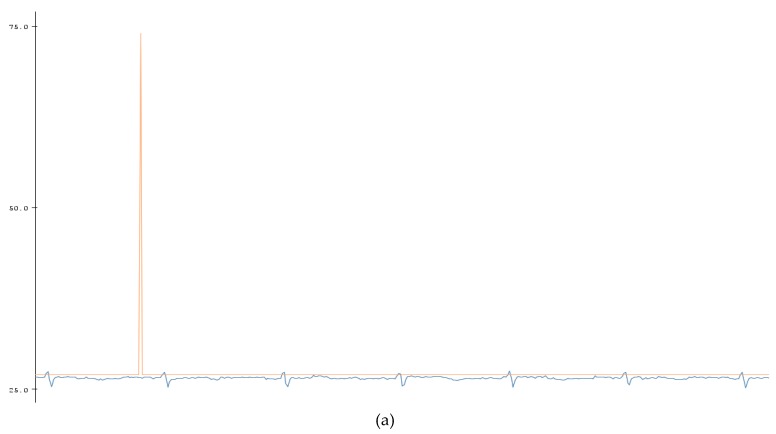

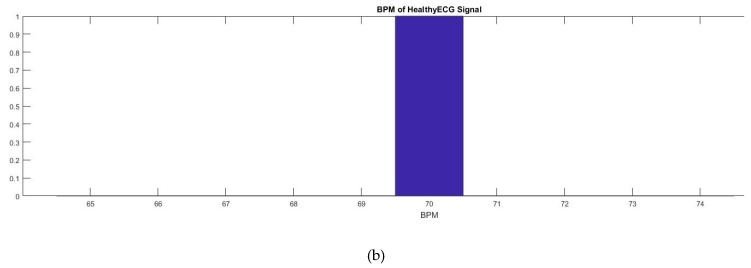
(**a**) Signal acquired from Bitalino ECG sensor and Arduino serial monitor (Red Spike: beats per minute (BPM), Blue: ECG signal. (**b**) BPM data plotted in Matlab (70 BPM).

**Figure 7:**
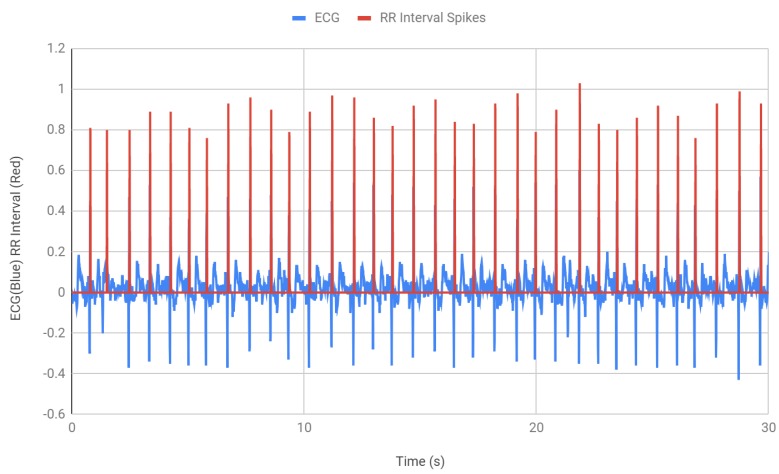
RR interval (data obtained using Bitalino ECG sensor and Arduino microcontroller).

A BPM counter was also added into the code which would allow someone to view the heart rate at a specific time (Figure 6). As shown in Figure 7, this was mainly completed by the use of the millis () function used by Arduino. The millis () Arduino function acted as a timer which started once the Arduino was initialized and continuously counts until the Arduino is turned off or manually reset. With that knowledge, currentMillis, previousMillis, and interval variables were created and used as a resetting timer to count the BPM. The variable “currentMillis” monitored the current time of millis (), “previousMillis” would monitor the starting time in millis () when each interval started. The variable “interval” was the time interval of the BPM (Table 1). The interval was set to 60,000 ms or 60 s. The variable “counter” counted the highest peak of the ECG signal which was the QRS peak to give the BPM. After the BPM was found, the counter was reset. To make sure that the Arduino code can easily count the peaks, the BPM counter was set to the peak amplitude threshold which is shown by a horizontal red line in Figure 6a. That way any peak that was intersected by the horizontal line, the peak threshold, would be counted by the device. The red spike in Figure 6a is the BPM and shows the total peak number counted by the code. In this case, the BPM was 70 which was within the healthy parameter. The data was then plotted in Matlab as shown in Figure 6b.

After finding the BPM, the next step was to detect the QRS waves and find the interval between two consecutive R peaks. Figure 7 shows the measurement of the RR intervals. An “if” statement was incorporated in the Arduino code that counts each R peak (Table 2). Within that if statement, the time was recorded and saved into a variable RR1. When the if statement was used again, for the next peak, the second time was recorded and the time difference between the peaks is recorded. The RR interval, RR1, which was the previous time, was determined by using the function millis () and then the current time that was stored as RR2 was subtracted from the previous time RR1 to get the RR interval. In Figure 7, the red spikes demonstrate the QRS peaks that were being monitored and the time interval between each peak. All the intervals were found to be within the healthy RR interval parameter 0.6–1.2 s. The intervals were then stored in RRadd which consequently collected the RR intervals for 30 s of ECG.

After successfully determining the BPM and RR intervals from the acquired ECG signal, the next step was to run a sanity check, which means the system should recognize if a person is healthy or not and if there is any abnormal cardiac activities such as rapid or inconsistent heart rate, the device should be able to inform via showing an alert. Two separate methods were tested for the alarm. The first method required observation of each and every RR interval. The second method involved finding the percent relative range. For every 30 s, the difference between the RR max and RR min interval values were found and then divided by the average RR interval value to find RRmax Percentage and RRmin Percentage. This was required to demonstrate how close the maximum and minimum values were to the average value. Table 3 shows some of the maximum and minimum percentages obtained from the 300 s interval. The max RR values were found to be about 98.5%–100% close to the average value and the min RR values were 96%–98.5% close to the average value, which proves the regularity between the RR intervals and thus the a consistent healthy heart rate.

**Figure 8:**
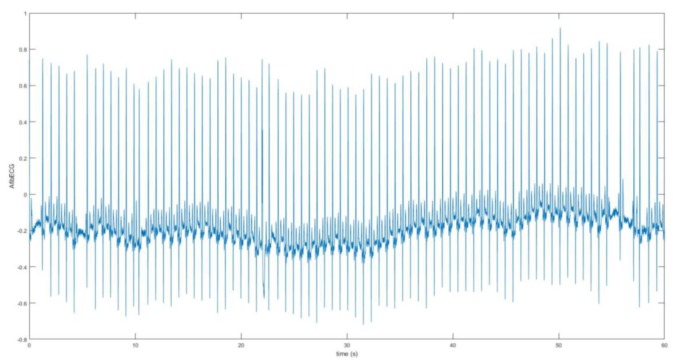
Atrial fibrillation (AF) ECG signal for 60 s (data obtained from the MIT-BIH database).

**Figure 9:**
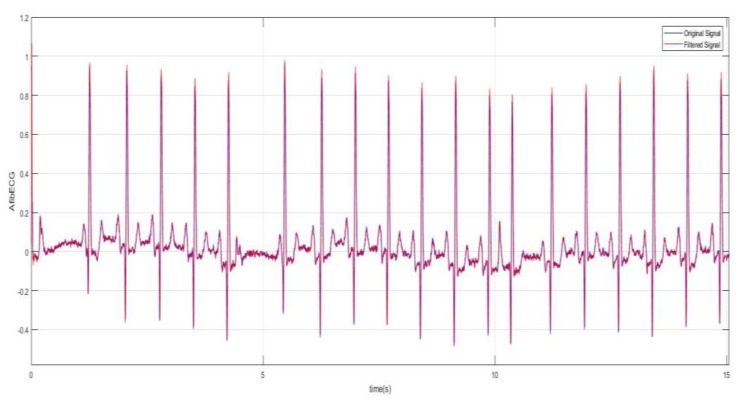
AF filtered ECG signal plotted for 15 s (data obtained from the MIT-BIH database).

**Figure 10:**
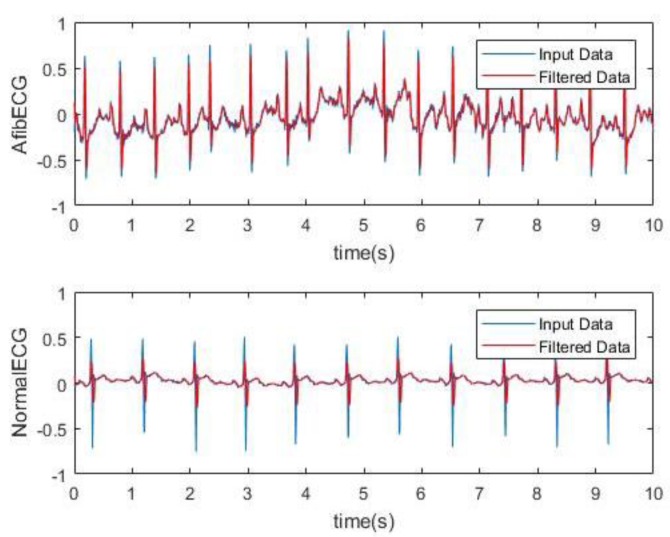
Comparison of characteristics of AF and healthy ECG signals (10 s).

The second part of this research work involves the acquisition and analysis of the unhealthy ECG signal with AF features. The AF ECG signal data was obtained from the MIT-BIH Atrial Fibrillation Database, which includes 25 long-term ECG recordings of human subjects with AF disorder. The signal was chosen based on better quality from these recordings. The data was loaded onto Matlab. The raw signal was converted into real unit signal by subtracting the base value from the signal and then dividing by the gain value. Following this, using the sampling frequency which was 250 Hz and the length of the signal, the time vector was determined for the plot. Figure 8 shows the AF ECG signal plotted over 60 s. Figure 9 better demonstrates the irregularity and the inconsistency of the abnormal ECG as the data was plotted for 15 s. The moving-average filter was also used here to smoothen the data as discussed previously. Next, plots in Figure 10 show how significantly AF ECG signals differ from the normal ECG signals. 

**Figure 11:**
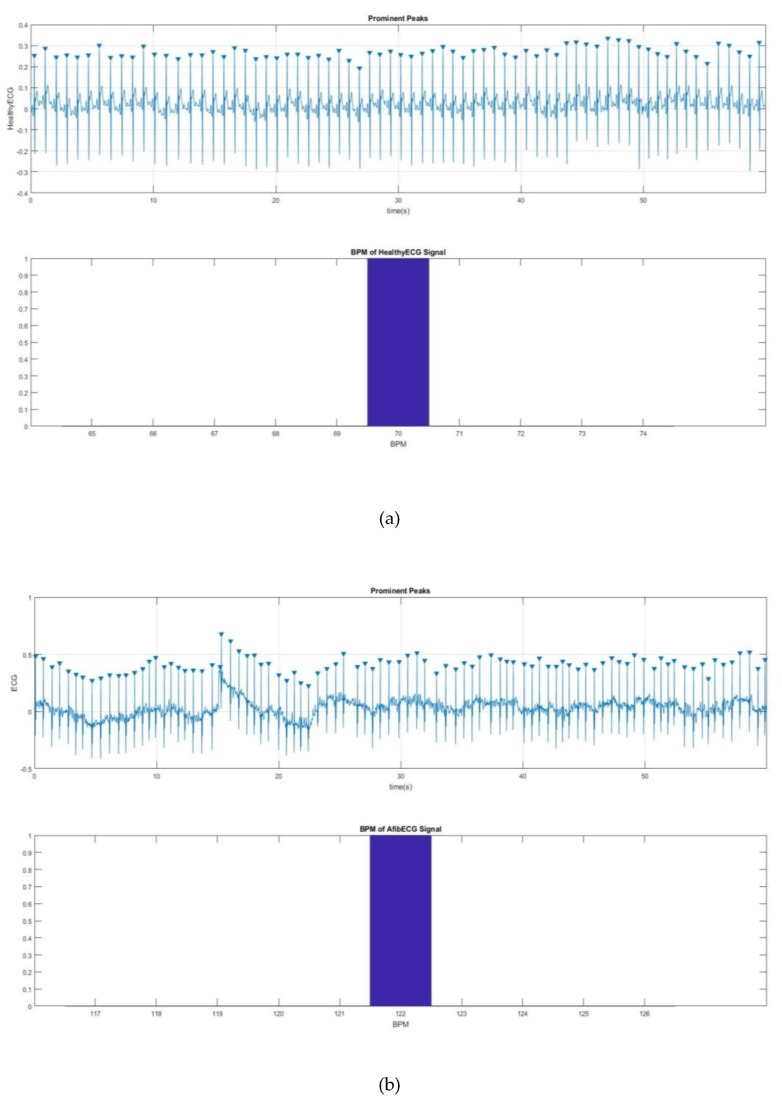
(**a**) 70 BPM for healthy ECG and (**b**) 122 BPM for AF ECG.

Next, the BPM for the AF ECG signal was determined. Figure 11 shows a way to detect the QRS peak and to find the BPM for the both healthy and AF ECG signals. Figure 11a,b shows 70 and 122 BPM, respectively, which also proves the rapid heart rate, one of the main characteristics of AF.

**Figure 12:**
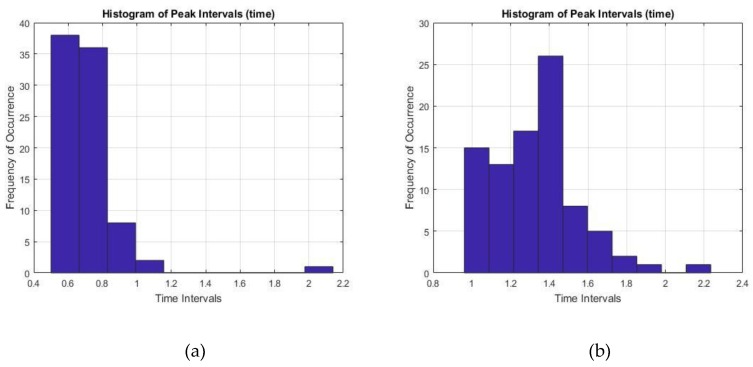
Histogram of RR peak intervals for (**a**) healthy ECG data and (**b**) AF ECG data.

Finally, the time interval between two prominent R peaks were monitored and recorded for 60 s. The RR interval values for both healthy ECG and the AF ECG were plotted as a histogram in Figure 12, where Figure 12a showed pretty consistent healthy ECG data and also within the healthy parameter of 0.6–1.2 s. On the other hand, Figure 12b showed inconsistent intervals of AF ECG data not within the healthy range which is another important characteristic of AF disorder.

**Figure 13:**
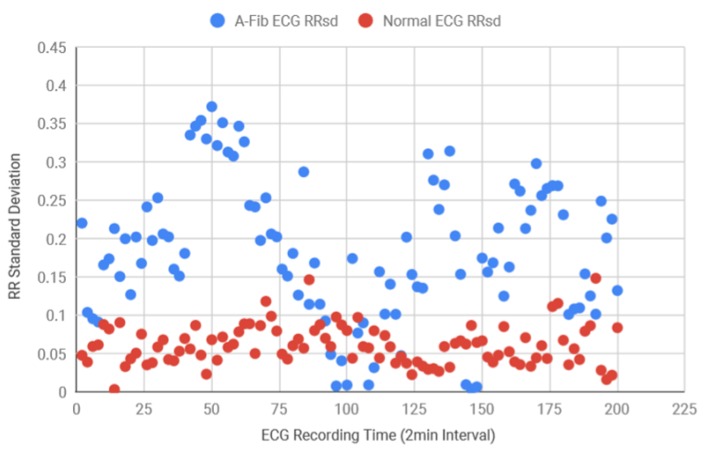
Variance in standard deviation for RR interval data collected from multiple subjects.

**Figure 14:**
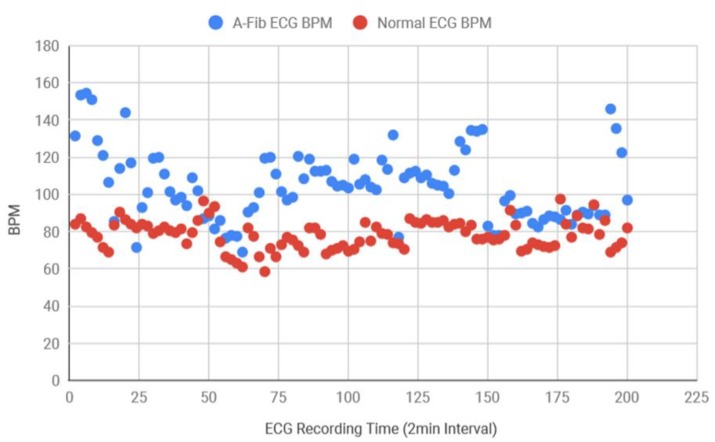
Variance in BPM data collected from multiple subjects.

Following this, for the diagnosis purposes and also to determine the accuracy of the system, both AF and normal ECG recordings from eight different subjects were collected in 120 s intervals from the MIT-BIH database. Using the Matlab function, the standard deviation of the RR intervals was calculated and the scattered plot was created in Figure 13 which demonstrates that AF data consistently had more variance in RR intervals in comparison to the healthy data. Healthy standard deviations were found to be significantly lower than the AF data. Since the majority of the standard deviation data for AF ECG were 0.12 above, the threshold value for the detection algorithm was determined as 0.12. Next, the BPM for AF and healthy ECG signals were also determined and plotted in Figure 14, where the healthy BPM were below 100 and mostly around 80, whereas the AF ECG BPM were significantly higher than the healthy ECG. The pattern for AF BPM was expected to be higher than 100. However, a good amount of BPM data was found to be around 90–100.

**Figure 15:**
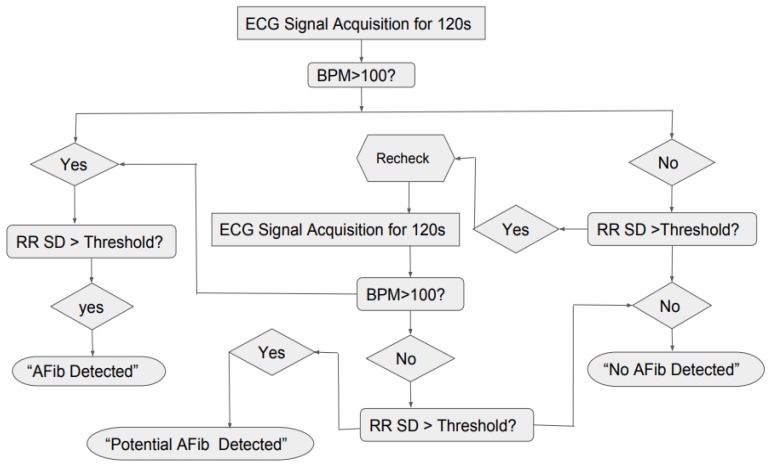
Detection logic diagram.

Based on the results for standard deviation and the BPM shown in Figure 13 and Figure 14, the general logic for the AF detection was created in Figure 15, where the system would take the ECG reading for 120 s, then it would check if the BPM is greater than 100 and RR SD greater than the threshold or not. If the both the conditions were true, the program would show “AF Detected” and if both the conditions were false the program would show “No AF Detected”. However, since it was found that for AF ECG, the BPM can sometimes be lower than 100 also, the second confirmation factor was added to the logic, where the system would recheck the ECG for 120 s and repeat the previous steps. If the BPM was found to be less than 100 but standard deviation was greater than the threshold, the program would show “Potential AF Detected.”

**Figure 16:**
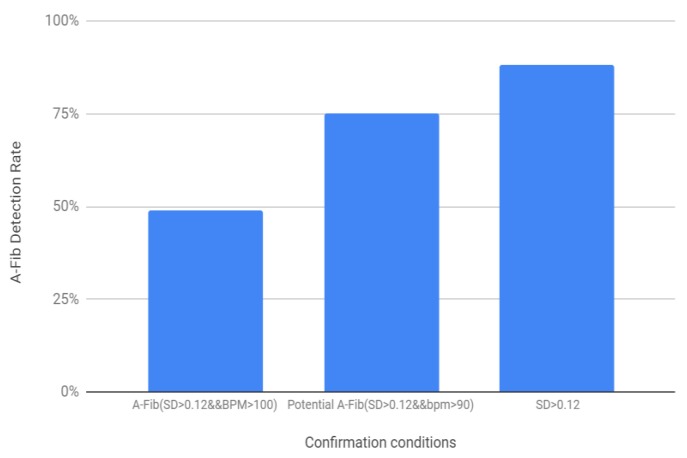
Detection rates for various threshold conditions.

Based on the above-mentioned threshold values and the logic diagram in Figure 16, the success rate for the AF detection was determined for the 200 samples (100 healthy and 100 AF) (Figure 16). For the first condition, where SD > 0.12 and BPM > 100 showed 49% success rate, when the BPM threshold was lowered to 90 the success rate was 75%. The success rate for AF detection was found to be the highest, 88% when the second condition was added to determine the potential AF detection.

## 4. Discussion

At the very beginning of this project, consistent issues were observed regarding the acquisition of a proper and consistent ECG signal, which were mostly due to loose wiring of the experimental setup or the power interference. Additionally, while placing the electrodes, better ECG signals were obtained when the positive electrode was placed on the right wrist, the negative on the left wrist, and when the reference electrode was excluded from the system. After achieving the acceptable signal, digital filtering was applied to help smooth the curve so that the P wave could be seen. The RMS value function seemed to slightly smoothen the curve with 10 iterations and more iterations could possibly smooth the curve even further. However, the moving-average filter seemed to smoothen the signal significantly. Since detecting the P wave in AF ECG can be very tricky, this research work mainly focused on the QRS complex wave, which is the most prominent feature of the ECG signal as well as the least sensitive to muscle movement. The BPM code was first tested via the pseudo code and then with the sensor. During counting the peaks, it was difficult to determine what peaks were monitored and counted by the code, since some of the peaks were too low. To make it easily detectable, a horizontal red line was added to the code as reference. The RR interval code was then developed and tested to analyze the consistency of the heart rate. However, the algorithm could not be tested using the sensor first, due to some unknown issues with the sensor. After rewiring the whole setup and successfully achieving a better and consistent signal, the RR interval program was tested. The program was able to detect each and every RR interval between 0.6–1.2 s and with the BPM within 60–100. Therefore, the code was able to record the RR and verify if the intervals were within the healthy parameters. If the intervals were outside the parameters it would record that, and if five unhealthy RR intervals were recorded, the code would print “Alarm” for 5 s on the Arduino serial monitor and then reset the counter. Following this, the total RR intervals were monitored and stored in RRadd with a purpose of determining the RR average. After that, the minimum and the maximum RR values were determined in percentages to see the similarity with the average RR. It was found that the maximum and minimum intervals were 96%–100% close to the average interval. This might be a better method for deciding if medical attention is needed. If the Max and Min percentages tend to differ by a significant amount from the average RR values that will demonstrate the inconsistency in heart rate. Further study and testing may prove if specific percentage readings are adequate to decide the abnormalities in the ECG and if medical attention is necessary. The AF ECG signal was obtained from the MIT-BIH online database since trials could not be done on a subject with atrial fibrillation concern. Therefore, for the validation purpose and to show this system could be a feasible solution, the data was fed into the code in a similar way the Bitalino sensor and the Arduino microcontroller would carry out data acquisition. The obtained results showed similarity with the information found from the background research.

The experimental setup and the developed program using the Bitalino sensor and the microcontroller successfully determined BPM and RR interval within expected range for the healthy ECG, showed regularity in the heart rate, and the alarm function was also tested which showed no alert on the Arduino serial monitor for healthy subjects. Additionally, several ECG signals both with AF and healthy rhythm from different human subjects were collected from the MIT-BIH database and analyzed. The standard deviation for RR intervals were determined, and the RR SD plot showed more variance in AF data which proves inconsistency and irregularity in the RR intervals. The BPM plot demonstrates that AF BPM were mostly on the higher side and the BPM can reach up to 100–175, but not always, which supports the background research. The first condition, with SD > 0.12 threshold and BPM > 100 threshold, produced a relatively lower success rate, however, the success rate increased when the BPM threshold was lowered to 90. The success rate was found to be the highest when a second confirmation condition was added which was if at least RR SD is greater than the threshold or not. Therefore, based on the result analysis and AF detection success rate, inconsistent RR interval was found to be the most promising factor.

Comparing with the existing methods [22,23,24,25,26,27,28,29] to detect AF, the proposed method does not require complex numerical tools such as RdR maps [22] or Receiver Operating Characteristic (ROC) curves [25,28]. It also does not require computationally intensive algorithms [23,27] or probability-based data adaptive techniques [24], community-based screening [25], or mobile phone apps [29]. Instead, this research work focused on developing a noninvasive method to detect AF in a home setting based on low cost data acquisition hardware and low demand for computing power. 

## 5. Conclusions

The ultimate goal of this research work was developing a noninvasive low-cost method to detect AF in a home setting based on BPM and RR interval of ECG signal. Although we were not able to test the prototype device in real time on human subjects due to the limitation of access to human subjects with underlying AF conditions, the enabling technologies of such a low-cost device were tested and validated. This will allow the user with little or no knowledge about how to evaluate ECG signal or other related factors get a simple notification about potential AF concern and required health checkup. Even though this heart monitoring system is not a direct replacement as a standard ECG, the utilization of this affordable and user-friendly technological system at home will contribute towards reducing strokes, raising heart health awareness, and saving costs significantly. Basically, this device could be a prospective low-cost solution for AF detection in real time. Although this research provides a preliminary design, and testing method which showed promising results, there is scope to achieve more advancements with continued research such as (1) replacing the Arduino microcontroller with a microprocessor; (2) replacing the Bitalino sensor module with a commercial ECG sensor; (3) designing a compact signal conditioning circuit board, and (4) testing the algorithm on human subjects using proper statistical analysis

## Figures and Tables

**Table 1:** The Measurement subroutine used to calculate the BPM.

void Measurements(){ currentMillis=millis(); if (currentMillis-previousMillis>=interval) { previousMillis = currentMillis; RRavg = RRadd/counter; RRmaxPercent = ((RRmax-RRavg)/RRavg)*100; RRminPercent = ((RRmin-RRavg)/RRavg)*100; BPM = counter*2; counter = 0; RRadd = 0; RRmax = 0; RRmin = 0; } }

**Table 2:** The CountPeaks() subroutine and RRinterval() subroutine used to calculate the RR interval.

void CountPeaks(){ newvalue=(ECG>=0.35); if (newvalue==0){flag=1;} if (newvalue==1&&flag==1){ counter++; flag=0; RR1=millis(); RR=(RR1-RR2)/1000; } } void RRinterval(){ if(counter==1){RRmax=RR; RRmin=RR;} else{ if(RR>RRmax){RRmax=RR;} if(RR<RRmin){RRmin = RR;} RRadd = RRadd+RR; } }

**Table 3:** Percentage relative range for RR intervals.

RRmax (%)	RRmin (%)	Time (s)
98.77	97.22	30
99	97.51	60
99.9	96.22	90
98.72	98.38	120
98.98	96.14	150
98.77	97.44	180
98.85	97.33	210
98.76	97.09	240
99.01	96.79	270
98.86	97.33	300
